# Different clinical characteristics and treatment strategies for patients with localized sinonasal diffuse large B cell lymphoma and extranodal NK/T cell lymphoma

**DOI:** 10.1186/s13045-016-0368-9

**Published:** 2017-01-05

**Authors:** Yu Huang, Bo Jia, Shiyu Jiang, Shengyu Zhou, Jianliang Yang, Peng Liu, Lin Gui, Xiaohui He, Yan Qin, Yan Sun, Yuankai Shi

**Affiliations:** Department of Medical Oncology, Beijing Key Laboratory of Clinical Study on Anticancer Molecular Targeted Drugs, National Cancer Center/Cancer Hospital, Chinese Academy of Medical Sciences and Peking Union Medical College, No. 17 Panjiayuan Nanli, Chaoyang District, Beijing, 100021 China

**Keywords:** Diffuse large B cell lymphoma, Extranodal NK/T cell lymphoma, Sinonasal, Localized

## Abstract

The difference in clinical features and treatment outcomes between localized sinonasal diffuse large B cell lymphoma (SN-DLBCL) and sinonasal extranodal NK/T cell lymphoma (SN-ENKTL) is unclear. Therefore, we analyzed a total of 47 patients with localized SN-DLBCL and 211 patients with localized SN-ENKTL. The age distribution for these two subtypes is very distinct and the B symptoms were more common in SN-ENKTL. However, both SN-DLBCL and SN-ENKTL patients could achieve high overall response rate (ORR) and favorable prognoses. The 3-year overall survival (OS) rates for patients with SN-DLBCL and SN-ENKTL were 79.7 and 83.6% (*p* = 0.707), and the 3-year progression-free survival (PFS) rates were 61.4 and 70.1% (*p* = 0.294), respectively. For SN-DLBCL patients, chemotherapy followed by involved-field radiotherapy (IFRT) resulted in higher OS (83.7 vs 62.5%) and PFS (63.9 vs 50.0%) compared with chemotherapy alone, but the difference was not significant. No significant difference was found in the OS or PFS between radiotherapy alone and radiotherapy combined with chemotherapy for all patients with SN-ENKTL. But in extensive stage I and stage II SN-ENKTL patients, radiotherapy combined with chemotherapy could significantly improve the PFS (73.8 vs 50.0%) compared with radiotherapy alone. These results indicate that remarkable clinical disparities exist between localized SN-DLBCL and SN-ENKTL. However, different treatment strategies for them can result in similarly favorable prognoses.

**Electronic supplementary material:**

The online version of this article (doi:10.1186/s13045-016-0368-9) contains supplementary material, which is available to authorized users.

Dear Editor

Remarkable differences exist in the distribution of lymphoma subtypes between China and western populations. The incidence of extranodal NK/T cell lymphoma (ENKTL) is much higher in China [1, 2]. Diffuse large B cell lymphoma (DLBCL) and ENKTL are most common subtypes of sinonasal lymphomas. What is more, both sinonasal DLBCL (SN-DLBCL) and sinonasal ENKTL (SN-ENKTL) are typically diagnosed in the localized stage which represents 70–90% of cases [3, 4]. Since the difference between localized SN-DLBCL and SN-ENKTL has seldom﻿ been demonstrated before and is not clear, this study provides a comprehensive evaluation that focuses on clinical features and prognoses of localized SN-DLBCL and SN-ENKTL in Chinese patients.

A total of 47 consecutive patients with localized SN-DLBCL and 211 patients with localized SN-ENKTL from 2000 to 2014 were compared at the Cancer Hospital of Chinese Academy of Medical Sciences and Peking Union Medical College, Beijing, China. The methods used in this study are detailed in Additional file 1. This study showed that the incidence of SN-ENKTL was much higher than that of SN-DLBCL in China, which was opposite to the situation in the USA [4]. The ratio of males to females was 1.14:1 in SN-DLBCL and 2.40:1 in SN-ENKTL. Patients with SN-DLBCL had a higher median age (63 years old) than those with SN-ENKTL (40 years old). The B symptoms were more common in SN-ENKTL. The comparison of the main clinical characteristics between these two groups is presented in Table 1.

**Table 1:** Clinical characteristics of patients with localized SN-DLBCL and SN-ENKTL

Characteristic	All patients (*n* = 258), *n* (%)	SN-DLBCL (*n* = 47), *n* (%)	SN-ENKTL (*n* = 211), *n* (%)	*p*(SN-DLBCL vs. SN-ENKTL)
Sex				
Male	174 (67.4)	25 (53.2)	149 (70.6)	0.021
Female	84 (32.6)	22 (46.8)	62 (29.4)	
Age (year)				
Median (range)	43 (10–85)	63 (11–82)	40 (10–85)	<0.001
≤60	225 (87.2)	25 (53.2)	200 (94.8)	
>60	33 (12.8)	22 (46.8)	11 (5.2)	
Modified Ann Arbor stage				
Limited I	73 (28.3)	3 (6.4)	70 (33.2)	0.001
Extensive I	126 (48.8)	32 (68.1)	94 (44.5)	
II	59 (22.9)	12 (25.5)	47 (22.3)	
Nodal involvement				
Present	49 (19.0)	10 (21.3)	39 (18.5)	0.659
Absent	209 (81.0)	37 (78.7)	172 (81.5)	
B symptoms				
Present	104 (40.3)	5 (10.6)	99 (46.9)	<0.001
Absent	154 (59.7)	42 (89.4)	112 (53.1)	
LDH level				
Normal	192 (74.4)	37 (78.7)	155 (73.5)	0.514
Elevated	64 (24.8)	10 (21.3)	54 (25.6)	
Unknown	2 (0.8)	0 (0.0)	2 (0.9)	
ECOG performance status				
0	141 (54.7)	25 (53.2)	116 (55.0)	0.018
1	94 (36.4)	13 (27.7)	81 (38.4)	
≥2	23 (8.9)	9 (19.1)	14 (6.6)	
mIPI				
0	126 (48.8)	13 (27.7)	113 (53.6)	0.001
1	92 (35.7)	20 (42.5)	72 (34.1)	
2–4	38 (14.7)	14 (29.8)	24 (11.4)	
Unknown	2 (0.8)	0 (0.0)	2 (0.9)	
Treatment strategy				
CMT	147 (57.0)	37 (78.7)	110 (52.1)	<0.001
Chemotherapy alone	17 (6.6)	10 (21.3)	7 (3.3)	
Radiotherapy alone	94 (36.4)	0 (0.0)	94 (44.6)	
Response to treatment				
CR/CRu	205 (79.5)	36 (76.6)	169 (80.1)	0.686
PR	23 (8.9)	6 (12.7)	17 (8.1)	
SD	1 (0.4)	0 (0.0)	1 (0.5)	
PD	15 (5.8)	2 (4.3)	13 (6.2)	
Not evaluable	14 (5.4)	3 (6.4)	11 (5.1)	

*SN-DLBCL* sinonasal diffuse large B cell lymphoma, *SN-ENKTL* sinonasal extranodal NK/T cell lymphoma, *LDH* lactate dehydrogenase, *ECOG* Eastern Cooperative Oncology Group, *mIPI* modified International Prognostic Index, *CMT* combined modality therapy, *CR* complete response, *CRu* unconfirmed complete response, *PR* partial response, *SD* stable disease, *PD* progressive disease

The overall response rates (ORR) after completion of therapy were equally high for patients with SN-DLBCL and SN-ENKTL (89.3 vs 88.2%). For patients with SN-DLBCL, the ORR was 94.6% after chemotherapy followed by involved-field radiotherapy (IFRT) and 70% after chemotherapy alone. For patients with SN-ENKTL receiving radiotherapy combined with chemotherapy (combined modality therapy, CMT) and radiotherapy alone, the ORR were 91.8 and 87.2%. In CMT SN-ENKTL patients, the ORR and complete response (CR) rates were both 100% for chemotherapy containing pegaspargase or gemcitabine combined with radiotherapy group. In contrast, the ORR and CR rates were 87.1 and 79.0% for conventional chemotherapy regimens (cyclophosphamide, doxorubicin, vincristine, and prednisone, CHOP/CHOP-like) combined with radiotherapy group. In addition, the ORR was only 42.9% for patients with SN-ENKTL receiving chemotherapy alone which mainly consists of CHOP or CHOP-like regimens.

The treatment outcomes were similar between SN-DLBCL and SN-ENKTL. The 3-year overall survival (OS) and progression-free survival (PFS) rates were 79.7 and 61.4% for SN-DLBCL and 83.6 (*p* = 0.707) and 70.1% (*p* = 0.294) for SN-ENKTL, respectively (Fig. 1). Unfavorable Eastern Cooperative Oncology Group (ECOG) performance status (PS) and failure to achieve CR were significantly associated with worse OS and PFS for SN-DLBCL patients (Additional file 2). For patients with SN-ENKTL, factors related to worse OS and PFS included unfavorable modified Ann Arbor stage and failure to achieve CR (Additional file 2). In addition, higher modified International Prognostic Index (mIPI) [5] was significantly associated with worse OS but not significantly associated with worse PFS (Additional file 2).

**Fig. 1:**
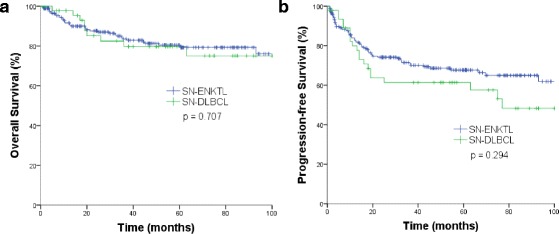
**a** Overall survival and **b** progression-free survival for patients with localized sinonasal diffuse large B cell lymphoma (SN-DLBCL, *n* = 47) and sinonasal extranodal NK/T cell lymphoma (SN-ENKTL, *n* = 211)

Although a short course of rituximab-CHOP (R-CHOP) chemotherapy followed by IFRT is the recommended treatment option for localized DLBCL, the treatment strategy for SN-DLBCL has seldom been explored because of limited cases. Lee et al. [6] found no significant differences in response rate and OS between patients with SN-DLBCL treated with R-CHOP chemotherapy alone and those treated with R-CHOP chemotherapy followed by IFRT. In our study, some patients were treated with CHOP or CHOP-like regimens without rituximab; the 3-year OS rates were 83.7% for patients receiving chemotherapy followed by IFRT and 62.5% for those receiving chemotherapy alone, but the difference was not significant (*p* = 0.113, Additional file 3).

Because of their different chemosensitivities, the treatment options for patients with ENKTL differ from those for patients with DLBCL. Currently, the optimal combination treatment modalities for localized SN-ENKTL have not been defined. Treatment options include radiotherapy alone, sequential chemotherapy and radiotherapy, or concurrent chemoradiotherapy [7, 8]. In this study, no significant difference was found in the OS or PFS between radiotherapy alone and CMT for all patients with localized SN-ENKTL (Additional file 4). But in extensive stage I and stage II SN-ENKTL group, CMT could significantly improve the PFS (73.8 vs 50.0%, *p* = 0.003) compared with radiotherapy alone, and CMT achieved higher 3-year OS rate though the difference was not significant (Additional file 4). In all SN-ENKTL patients receiving the CMT, chemotherapy containing pegaspargase or gemcitabine could achieve higher 3-year OS and PFS rates than conventional CHOP/CHOP-like chemotherapy regimens, but the difference was not significant.

In conclusion, the data from our study revealed that localized SN-DLBCL and SN-ENKTL had different clinical characteristics, but both of these two subtypes could achieve favorable prognoses. These results highlight that the heterogeneity of sinonasal lymphomas and therapeutic approaches should be selected according to the specific subtype and the stage at diagnosis.

## Additional files

Additional file 1: Supplemental methods. (DOCX 12 kb)
Additional file 2: Univariate analysis of prognostic factors for patients with localized SN-DLBCL and SN-ENKTL. (DOCX 19 kb)
Additional file 3: Treatment outcomes for patients with localized SN-DLBCL. (A) Overall survival and (B) progression-free survival of chemotherapy followed by involved-field radiotherapy (IFRT, *n* = 37) and chemotherapy alone (*n* = 10). (DOCX 40 kb)
Additional file 4: Treatment outcomes for patients with localized SN-ENKTL. (A) Overall survival and (B) progression-free survival for all SN-ENKTL patients (*n* = 211) treated with combined modality therapy (CMT) and radiotherapy alone. (C) Overall survival and (D) progression-free survival for extensive stage I and stage II SN-ENKTL patients (*n* = 141) treated with combined modality therapy (CMT) and radiotherapy alone. (DOCX 83 kb)

## Abbreviations

CHOP: Cyclophosphamide, doxorubicin, vincristine, and prednisone
CMT: Combined modality therapy
CR: Complete response
CRu: Unconfirmed complete response
ECOG: Eastern Cooperative Oncology Group
IFRT: Involved-field radiotherapy
LDH: Lactate dehydrogenase
mIPI: Modified International Prognostic Index
ORR: Overall response rate
OS: Overall survival
PD: Progressive disease
PFS: Progression-free survival
PR: Partial response
SD: Stable disease
SN-DLBCL: Sinonasal diffuse large B cell lymphoma
SN-ENKTL: Sinonasal extranodal NK/T cell lymphoma
