# Supplementary material for: Different clinical characteristics and treatment strategies for patients with localized sinonasal diffuse large B cell lymphoma and extranodal NK/T cell lymphoma
Source: J Hematol Oncol. 2017 Jan 5;10:7. doi: 10.1186/s13045-016-0368-9 (PMC5217200; doi:10.1186/s13045-016-0368-9)
Supplement: Additional file 1: — Supplemental methods. (DOCX 12 kb) [file 13045_2016_368_MOESM1_ESM.docx]

**Supplementary File 1.**

**Methods**

**Patients**

Between 2000 and 2014, 258 consecutive patients diagnosed with primary nasal cavity or paranasal sinus DLBCL and ENKTL were retrospectively reviewed at the Cancer Hospital of Chinese Academy of Medical Sciences and Peking Union Medical College, Beijing, China. The diagnoses for DLBCL and ENKTL were confirmed by pathological and immunohistochemical evaluations according to the World Health Organization classification. The modiﬁcation of the Ann Arbor system was used for staging. Limited stage ⅠE tumors were restricted to the nasal cavity or paranasal sinus, whereas extensive stage ⅠE tumors extended into the neighboring structures without any sign of nodal or distant dissemination. Since this study only consisted of patients with stage Ⅰ/Ⅱ disease, the modified International Prognostic Index (mIPI) was applied at the time of diagnosis in order to evaluate the prognostic significance. This study was approved by the Ethics Committee of Cancer Hospital, Chinese Academy of Medical Sciences.

**Treatment**

Chemotherapy was the primary treatment for DLBCL. All patients with localized stage SN-DLBCL received combination chemotherapy with or without IFRT. The chemotherapy consisted of cyclophosphamide, doxorubicin, vincristine, prednisone (CHOP) or CHOP-like regimens with or without rituximab. Overall, 37 patients received chemotherapy with IFRT, and 10 patients received chemotherapy alone. Radiotherapy was considered the primary treatment for localized patients with SN-ENKTL. 110 patients received the CMT and 94 patients were treated with radiotherapy alone. Only 7 patients received chemotherapy alone.

**Criteria for treatment outcomes**

The efficacy of treatment was evaluated using the international workshop response criteria for lymphomas. Progression-free survival (PFS) was defined as the time from the first day of treatment to the date of disease progression, re-currence, or death due to any cause. Overall survival (OS) was measured from the first day of treatment to the date of death or the date of last follow-up.

**Statistical Analysis**

Comparisons of clinical features and initial response rates were performed using the χ^2^ test for categorical variables. The PFS and OS were calculated using the Kaplan–Meier method, and comparisons between groups were made using log-rank tests. A *p* value less than 0.05 was considered as statistically significant.
