# Supplementary material for: Different clinical characteristics and treatment strategies for patients with localized sinonasal diffuse large B cell lymphoma and extranodal NK/T cell lymphoma
Source: J Hematol Oncol. 2017 Jan 5;10:7. doi: 10.1186/s13045-016-0368-9 (PMC5217200; doi:10.1186/s13045-016-0368-9)
Supplement: Additional file 2: — Univariate analysis of prognostic factors for patients with localized SN-DLBCL and SN-ENKTL. (DOCX 19 kb) [file 13045_2016_368_MOESM2_ESM.docx]

**Supplementary File 2.** Univariate analysis of prognostic factors for patients with Localized SN-DLBCL and SN-ENKTL

| Factors | SN-DLBCL | | | | SN-ENKTL | | | |
| --- | --- | --- | --- | --- | --- | --- | --- | --- |
|  | 3-y OS (%) | *p* | 3-y PFS (%) | *p* | 3-y OS (%) | *p* | 3-y PFS (%) | *p* |
| Modiﬁed Ann Arbor stage | | | | | | | | |
| Limited Ⅰ | NA | 0.698 | NA | 0.545 | 93.7 | 0.014 | 84.3 | 0.008 |
| ExtensiveⅠ | 79.8 |  | 58.8 |  | 79.8 |  | 63.2 |  |
| Ⅱ | 75.0 |  | 66.7 |  | 75.4 |  | 63.1 |  |
| ECOG performance status | | | | | | | | |
| 0-1 | 84.6 | 0.032 | 71.5 | 0.002 | 84.7 | 0.088 | 70.7 | 0.356 |
| ≥2 | 53.3 |  | 22.2 |  | 68.8 |  | 60.9 |  |
| mIPI | | | | | | | | |
| 0-1 | 84.2 | 0.207 | 63.6 | 0.853 | 85.2 | 0.016 | 72.2 | 0.071 |
| 2-4 | 70.1 |  | 57.1 |  | 69.4 |  | 61.3 |  |
| Response to treatment | | | | | | | | |
| CR/CRu | 96.6 | <0.001 | 77.1 | <0.001 | 91.1 | <0.001 | 82.8 | <0.001 |
| Others | 16.7 |  | 0.0 |  | 28.6 |  | 0.0 |  |

NA Not available for analysis due to small number of patients, SN-DLBCL sinonasal diffuse large B-cell lymphoma, SN-ENKTL sinonasal extranodal NK/T-cell lymphoma, OS overall survival, PFS progression-free survival, LDH lactate dehydrogenase, ECOG Eastern Cooperative Oncology Group, mIPI modified International Prognostic Index, CR complete response, CRu unconfirmed complete response
