# Supplementary material for: Different clinical characteristics and treatment strategies for patients with localized sinonasal diffuse large B cell lymphoma and extranodal NK/T cell lymphoma
Source: J Hematol Oncol. 2017 Jan 5;10:7. doi: 10.1186/s13045-016-0368-9 (PMC5217200; doi:10.1186/s13045-016-0368-9)

**Supplementary File 3.** Treatment outcomes for patients with SN-DLBCL.

(A) Overall survival of chemotherapy followed by involved-field radiotherapy (IFRT, n=37) and chemotherapy alone (n=10).


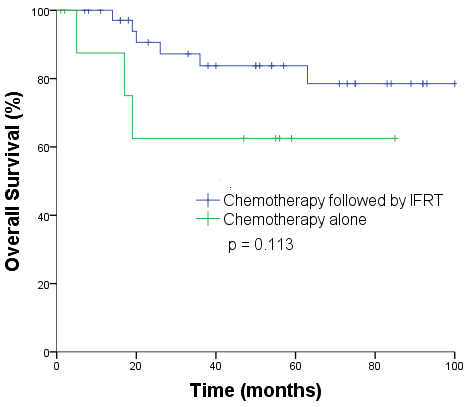


(B) Progression-free survival of chemotherapy followed by involved-field radiotherapy (IFRT, n=37) and chemotherapy alone (n=10).


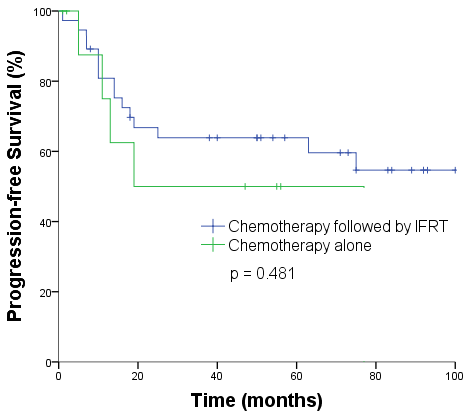

Supplement: Additional file 3: — Treatment outcomes for patients with localized SN-DLBCL. (A) Overall survival and (B) progression-free survival of chemotherapy followed by involved-field radiotherapy (IFRT, n = 37) and chemotherapy alone (n = 10). (DOCX 40 kb) [file 13045_2016_368_MOESM3_ESM.docx]
