# Supplementary material for: Different clinical characteristics and treatment strategies for patients with localized sinonasal diffuse large B cell lymphoma and extranodal NK/T cell lymphoma
Source: J Hematol Oncol. 2017 Jan 5;10:7. doi: 10.1186/s13045-016-0368-9 (PMC5217200; doi:10.1186/s13045-016-0368-9)

**Supplementary File 4.** Treatment outcomes for patients with localized SN-ENKTL.

(A) Overall survival for all SN-ENKTL patients (n=211) treated with combined modality therapy (CMT) and radiotherapy alone.


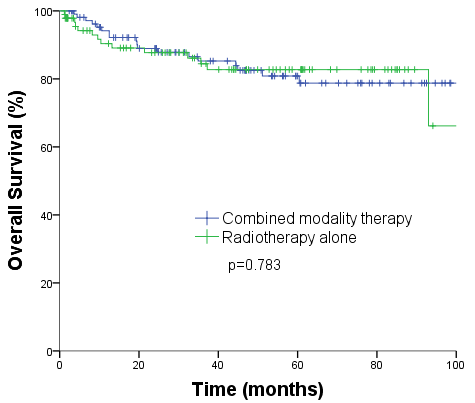


(B) Progression-free survival for all SN-ENKTL patients (n=211) treated with combined modality therapy (CMT) and radiotherapy alone.


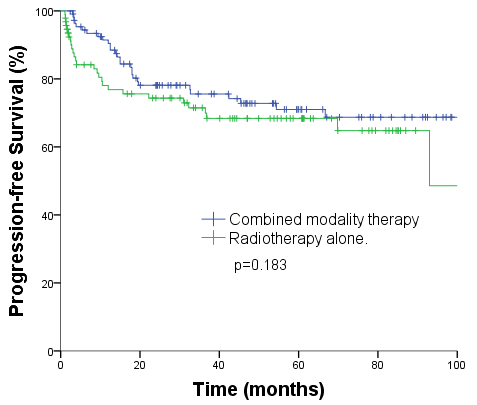


(C) Overall survival for extensive stage I and stage II SN-ENKTL patients (n=141) treated with combined modality therapy (CMT) and radiotherapy alone.


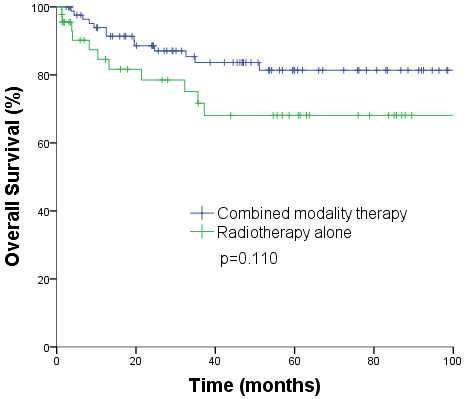


(D) Progression-free survival for extensive stage I and stage II SN-ENKTL patients (n=141) treated with combined modality therapy (CMT) and radiotherapy alone.


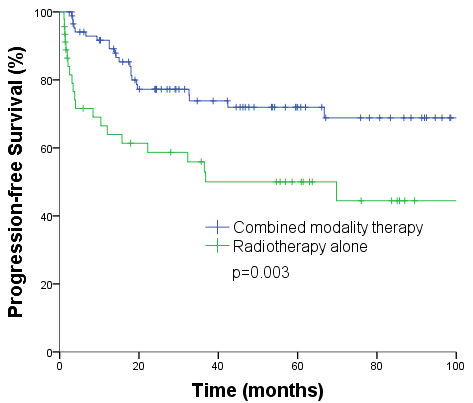

Supplement: Additional file 4: — Treatment outcomes for patients with localized SN-ENKTL. (A) Overall survival and (B) progression-free survival for all SN-ENKTL patients (n = 211) treated with combined modality therapy (CMT) and radiotherapy alone. (C) Overall survival and (D) progression-free survival for extensive stage I and stage II SN-ENKTL patients (n = 141) treated with combined modality therapy (CMT) and radiotherapy alone. (DOCX 83 kb) [file 13045_2016_368_MOESM4_ESM.docx]
